# Emerging Ecosystems Change the Spatial Distribution of Top Carnivores Even in Poorly Populated Areas

**DOI:** 10.1371/journal.pone.0118851

**Published:** 2015-03-23

**Authors:** Facundo Barbar, Victoria Werenkraut, Juan Manuel Morales, Sergio Agustín Lambertucci

**Affiliations:** Laboratorio Ecotono INIBIOMA (CONICET- Universidad Nacional del Comahue), Bariloche, Río Negro, Argentina; Dauphin Island Sea Lab, UNITED STATES

## Abstract

Humans affect biological diversity and species distribution patterns by modifying resource availability and generating novel environments where generalist species benefit and specialist species are rare. In particular, cities create local homogenization while roads fragment habitat, although both processes can increase food availability for some species that may be able to take advantage of this new source. We studied space use by birds of prey in relation to human construction, hypothesizing that these birds would be affected even in poorly populated areas. We worked in Northwestern Patagonia, Argentina, which is experiencing a high population growth, but still having very large unpopulated areas. We related the presence of raptors with different sources of human disturbance and found that both the abundance and richness of these birds were positively associated with anthropogenic environments. These results are driven mostly by a strong association between the medium-sized generalist species and these novel environments (mainly roads and cities). This may create an imbalance in intra-guild competitive abilities, modifying the normal structures of top carnivore hierarchies. Indeed, the structure of raptor communities seems to be changing, even in poorly populated areas, with anthropogenic constructions seemingly producing changes in wild areas more promptly than thought, a cause for concern in ecosystems conservation issues.

## Introduction

Anthropogenic activities and population growth are major causes of changes on biological diversity [1]. Human alterations on the environments have produced a decline in biodiversity and are elevating extinction rates of species at global scale [2]. However, biodiversity might be positively related to human population at a regional scale due, for instance, to an enhanced spatial heterogeneity between rural an urban environments, a new flux of energy and the introduction of exotic species [3–5]. Then, the influence of these modifications depends on both, the scale and the organisms involved, and conservation biologists must be aware of this [6].

The modification of habitats and availability of resources by humans create new emerging ecosystems [7,8]. These new ecosystems are characterized by spatial heterogeneity from areas with higher human modifications to the natural surrounding areas [9,10]. The consequent changes in the ecological functions may produce a new combination of species, sometimes modifying and, in many cases, increasing the local richness [8,11]. Two of the most important sources of these new environments are urbanization and transportation infrastructure [12,13].

Human settlements modify natural areas, reducing habitat available for some species and decreasing the local native diversity [14,15]. Moreover, communication ways as roads, and other infrastructures, such as power lines and fences generate habitat fragmentation [16]. These types of human disturbances tend to homogenize biotic diversity due to an association of some species which are better adapted to tolerate these changes [17]. Then, the emerging ecosystems can be advantageous or disadvantageous for different species depending on their life history, size, behavior, habitat perception and tolerance to human activities [18].

Generalists species are highly tolerant to human impacts, adapting themselves successfully to those environments, while habitat specialist are less adapted to cope with habitat alteration [19,20]. As a result of human activities specialist species of several taxa tend to decline globally [21–23]. This leads to a taxonomic and functional homogenization that affects directly the ecosystem services and ultimately the productivity and goods [24]. Functional homogenization due to the replacement of specialist by generalist species has been poorly studied even when it is a good estimator of biodiversity loss and alteration of the ecosystems [24,25].

Birds of prey are on the top of the food chain, showing a wide combination of trophic interactions and they are present both in pristine and altered environments [26–28]. Within this assemblage of birds, there are well recognized generalist species, with a wide niche and geographical distribution (e.g. the Chimango caracara [29,30]), and some specialists adapted to particular landscapes and diets (e.g. the Andean Condor [30,31]). Additionally, they are good biodiversity indicators of other taxa and can be used as surrogate species for accessing conservation issues and to identify environmental changes [26,32–34]. All these characteristics suggest that these bird species are suitable models to study the influence of human activities, even in relatively pristine habitats. The study of their patterns of abundance, richness and composition would allow us to assess possible processes of functional homogenization revealing a change in biodiversity due to human changes on the environment [28,35]. Our aim was to study the patterns of space use by birds of prey in relation to the presence of human constructions, under the hypothesis that raptors are affected by human made structures. Our prediction was that the subset of species considered as generalist will be more abundant in areas near to human constructions, while more specialized species will avoid these environments. For this, we studied the relationship between the presence, abundance and richness of raptors and the gradient of anthropic impact given by the distance from cities, human settlements, routes, and fences in the argentine Patagonia.

## Materials and Methods

### Ethics Statement

We did not collect or manipulate birds in this study. Permissions to conduct our censuses of birds of prey in the field were provided by Dirección de Fauna Silvestre de Río Negro and the owners and managers of local farms.

### Study area

The study was carried out in Northwestern Argentine Patagonia, in the proximities of San Carlos de Bariloche city (41°03´S—70°59´W; ca. 130,000 inhabitants). In this area human population is very low but the growth rate is among the most elevated in the country (density of people in Pilcaniyeu department where the study was carried out is about 0.70 p/km^2^) [36]. The landscape is a typical steppe area with open vegetation dominated by grasses (*Festuca pallescens, Stipa speciosa*) and shrubs (*Mulinum spinosum*), with an incursion of Andean Patagonian forest, dominated by Cordilleran cypress (*Austrocedrus chilensis*) [37]. The climate is cold-temperate (annual mean 6°C) and dry, with a mean annual precipitation of 800 mm. [38]. The area presents softly undulated hills with ridges and cliffs.

### Study Species and Sampling Methods

During austral later spring and summer of 2007–2008, we conducted stationary point counts, with a fixed observation radius and time (500 m and 30 min, modified from [39]). We registered all the raptor species and the number of individuals observed. The censuses were made by three observers to increase the chance of observing and identifying all the birds present in the plot, and also to avoid double counts of individuals (i.e., each observer followed the bird/s observed during the point count). We surveyed 77 different sites placed along 22 transects equidistant 1 km each, perpendicular to both sides of a primary road (N°23 National Road), and at different distances from the city. We did not include in any case pure urban environments, but sampled at the periphery of the city (around 6 km from the border). Within each transect, the first 3 points count were separated by 1 km each, starting from the road, and the last one, was separated 2 km from the third. All sites were censed between 2–12 times, depending on climatic conditions and accessibility. To avoid any difference due to time of the day, all censuses were completed during the morning (from one hour after sunrise to noon). We also avoided seasonal differences by conducting the censuses in the raptors reproductive season (October to March).

Thirteen raptors species live in this area: *Vultur gryphus, Coragyps atratus, Cathartes aura, Elanus leucurus, Circus cinereus, Parabuteo unicinctus, Geranoaetus melanoleucus, Buteo polyosoma, Caracara plancus, Milvago chimango, Falco peregrinus, F*. *femoralis* and *F*. *sparverius* [40]. All of the species observed (11) were used to estimate the richness; later on, we excluded seven species of the individual analysis due to their scarce records in our censuses and low detection probabilities modeled. The remaining six analyzed species were three obligated carrion eaters (*V*. *gryphus, C*. *atratus* and *C*. *aura*), two facultative carrion eaters (*C*. *plancus* and *M*. *chimango*) and one generalist hunter (*G*. *melanoleucus*) [40]. Those species were studied individually to estimate the effects of human made structures on their abundances.

### Anthropogenic variables

We characterized each site surveyed by measuring with GPS the distances to the nearest human constructions. We classified human made structures into five different types: 1) primary roads (2 vehicle lanes), 2) secondary roads (1 vehicle lane), 3) fences, 4) human settlements (i.e. few houses in the field), and 5) cities (more than 10,000 habitants).

### Data analysis

We used an occupancy framework [41,42] to evaluate the influence of the sources of human disturbances described above on species richness and abundance of individuals within single species. These models are a type of hierarchical models that allow estimating abundance and/or occurrence of a species corrected for imperfect detection using replicated sampling counts [42]. Data in the replicated counts arise from two distinct processes, one ecological and one observational. The ecological process describes the spatiotemporal variation in the imperfectly observed true state of the population. The observational process determines the data actually observed and is a representation of imperfect detection.

We modeled species richness using a Bayesian hierarchical multi-species occupancy approach (e.g.[43,44]). These models treat each species as a random sample from the studied community. Thus, an individual species´ response came from a common community-level distribution of responses. The ecological and observational processes are modeled as follow:

$$z_{i,k}∼Bern\left(\psi_{ik}\right)\;\text{Ecological process}$$

$$y_{i,j,k}|z_{i,k}∼Bern\left(p_{i,k}*z_{i,k}\right)\;\text{Observational process}$$

where the site-specific occupancy for site *i* = 1 to 77 and species *k* = 1 to 11 is an imperfectly observed Bernoulli random variable z_i,k_, where ψ_ik_ is the probability that species *k* occurs at site *i*. The latent variable z_i,k_ = 1 if species *k* occur at site *i* and is zero otherwise. The observed data y_i,j,k_ at site *i*, replicate *j* (*j* = 1 to 12), and for species *k* is conditional upon the true occurrence state (z_i,k_) and is also assumed to be a Bernoulli random variable if species *k* is present (i.e. if z_i,k_ = 1) where p_i,k_ is the probability of detecting the species *k* at site *i*; y_i,j,k_ is a fixed zero with probability 1 if species *k* is absent from site *i* (i.e. if z_i,k_ = 0). We also modeled a correlation (rho) between occurrence and detection because high abundance species are likely to be both easier to detect and more prevalent across the landscape [42,43].

We hypothesized that occurrence probability would vary by species and would be affected by distance to different sources of human disturbances (described above). We incorporated these effects in a linear model using a logit link as follows:

$$\text{logit}(\psi_{ik})=\alpha_{0k}+\beta_{1k}*{\text{primary roads}}_{i}+\beta_{2k}*{\text{secondary roads}}_{i}+\beta_{3k}*{\text{fences}}_{i}+\beta_{4k}*{\text{ human settlements}}_{i}+\beta_{5k}*\;{\text{cities}}_{i}$$

where α_0k_ is the occurrence probability for species *k* in sites with "average distance to human disturbances", and β_1k_, β_2k_, β_3k_, β_4k_, and β_5k_ are the coefficients for the effects of distance to primary roads_,_ secondary roads_,_ fences_,_ human settlements and cities for species *k*. These species-level coefficients were treated as random effects governed by community-level hyper-parameters. Thus, we assumed that for a given effect (e.g. distance to primary roads) the species level parameters came from a normal distribution described by community mean and standard deviation hyper-parameters (e.g. *β*_*1k*_ ∼ *N* (*μ*_*β*1_, *σ*_*β*1_)).

Species richness at each site was not directly modeled, but is a derived quantity based on the occurrence of individual species. Species richness at site *i* was calculated as $N_{i}=\sum_{k\;=\;1}^{11}z_{i,k}$. To evaluate the effect of different sources of human disturbance on species richness we compare the values of the community effects (hyper-parameters).

We estimated model parameters with software WinBUGS [45] using the package R2WinBUGS [46] to interface with R program [47]. We ran three parallel chains of 100,000 Markov Chain Monte Carlo (MCMC) iterations, we discarded the first half as burn-in and we kept 10,000 simulations. We used uninformative priors and random initial values. Convergence was assessed by visual inspection of MCMC chains and using the Gelman-Rubin statistic ("Rhat" [48]) with all diagnostic values <1.1 indicating convergence [48]. The full model specification is provided in Supporting Information S1 File.

To model the abundance of individuals within single species we used N-mixture models [49] as implemented in the *pcount* function of the package unmarked [50] in R software [47]. For abundance data, variability in the ecological process is usually modeled with a Poisson distribution as it is the natural candidate for describing animal abundance [51] but other distributions such as the negative binomial or a zero-inflated Poisson could be used to accommodate extra-Poisson variability [52]. The observational process is described by a binomial distribution with the true number of individuals and a detection probability as parameters. Here we fitted different N-mixture models for total raptor abundance, species richness, and for the abundance of individuals from the six raptor species listed above. For all models we accounted for local abundance over-dispersion using a negative binomial distribution. Thus, in their general form our N-mixture models were as follow:

$${\text{N}}_{i}∼\text{NegBin}\left(\lambda_{i},\alpha \right)\;\text{Ecological process}$$ (Eq. 1)

$$y_{i,j}∼\text{Binomial}\left(N_{i},p\right)\;\text{Observational process}$$ (Eq. 2)

where N_i_ is the local abundance at site *i*, which follows a Negative binomial distribution with mean λ and over-dispersion parameter α; y_i,j_ is the observed count at site *i* during replicate survey *j*, which is described by a binomial distribution with sample size N_i_ and detection probability p [49,53].

Here, we modeled local abundance at site *i* as function of the distance to different sources of human disturbances via logit-link functions as follow:

$$\text{log}\left(\lambda_{i}\right)=\alpha_{0}+\beta_{1}*{\text{primary roads}}_{i}+\beta_{2}*{\text{secondary roads}}_{i}+\beta_{3}*{\text{fences}}_{i}+\beta_{4}*\;{\text{human settlements}}_{i}+\beta_{5}*\;{\text{cities}}_{i}$$

where i = 1 to 77 and indicates the surveyed site, α_0_ is the intercept, β_1_, β_2_, β_3_, β_4_, and β_5_ are the site effects of the distance to primary roads_,_ secondary roads_,_ fences_,_ human settlements and cities respectively.

Before the analyses we standardized all predictors (mean = 0, standard deviation = 1), thus we were able to directly compare their relative explanatory power by means of their standardized coefficients [54]. We assumed that the community was closed over the two years during which the replicated surveys were conducted (i.e. the raptor pool remained constant). Also, the model assumes that the detection probability of individuals is constant for all species. We assumed this to be true because large birds are observed more easily, particularly in open areas, and raptors are in general medium-to-large sized birds, easy to be detected. Finally, we did not model the probability of the occurrence of additional species since we were able to register all species present in the area and there are not records of other species in previous publications (e.g.[30]).

## Results

We registered a total of 702 raptors in 436 point counts (77 sites), completing 218 hours of observation in 109 census days. We recorded every raptor species in the area, being *G*. *melanoleucus* the most abundant with 183 observations and *E*. *leucurus* the less abundant with only 2 observations in the same point count. The maximum number of individuals observed in one census was 29 birds of 4 different species, and the richest census had 5 different species with a total of 26 individuals.

**Fig 1:**
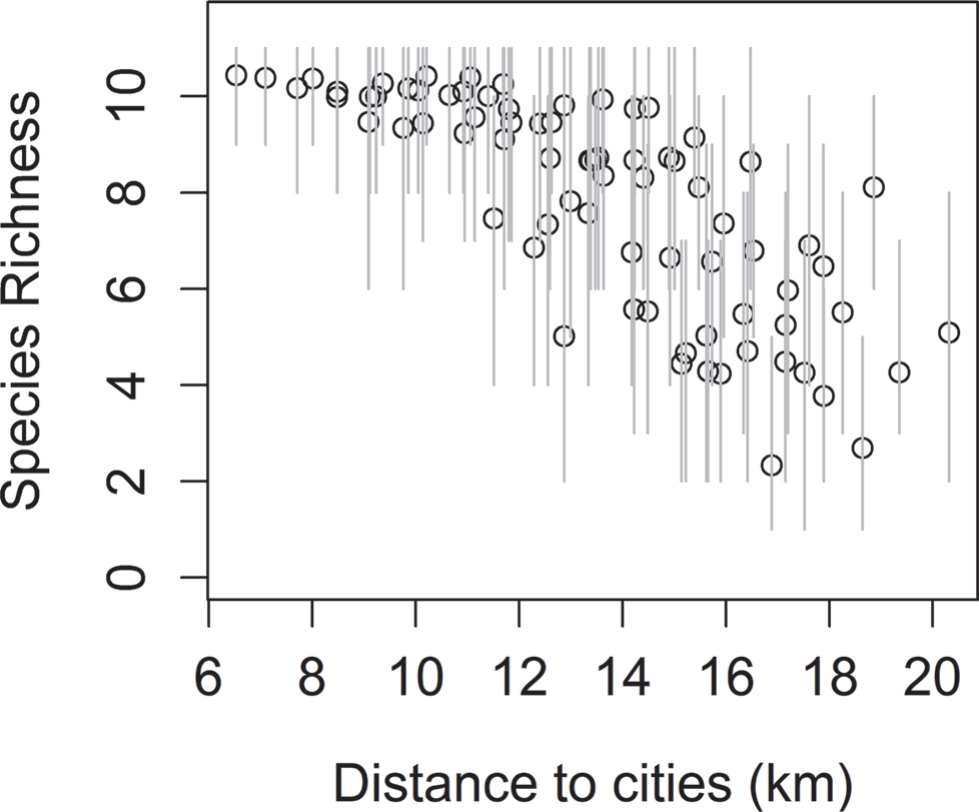
Relationship between the total richness of raptor species observed and the anthropogenic variable that was the most influential in our model.

Species richness increased near cities and fences, being the first the strongest predictor variable according to our model (Fig. 1, S1 Table).

**Fig 2:**
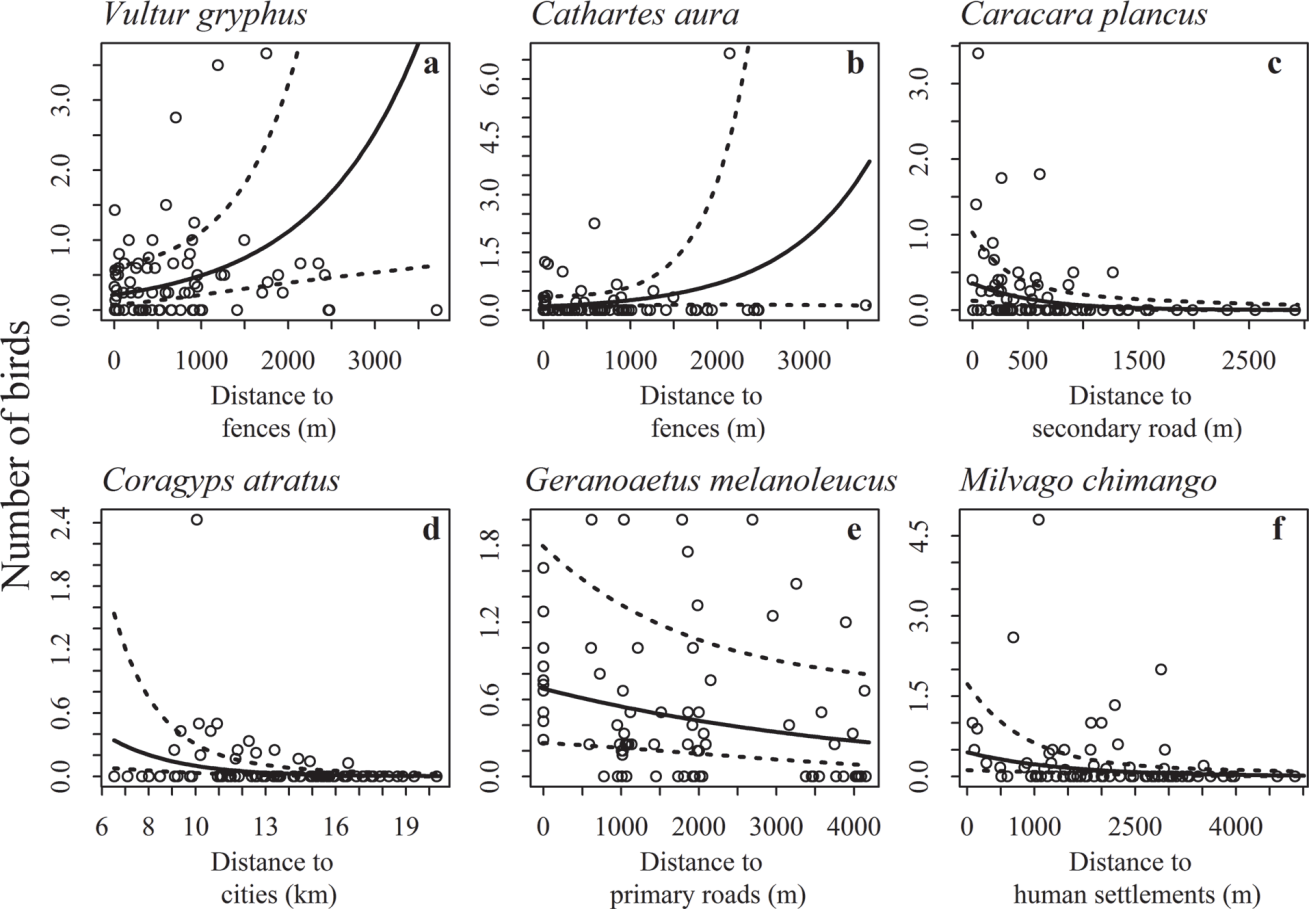
Relationship between each of the six more abundant raptor species and the anthropogenic variable that was most influential for that species in the models obtained.

After modeling the detection probability for each species under the N-mixture framework we analyzed the 6 which had similar values and less dispersion (Table 1). When we considered each species separately, we found that the proximity to human constructions had positive effects on the presence of most raptors (Table 2). Two species (*C*. *aura* and G. *melanoleucus*) lack of explanatory variables with strong significance, but both had one variable marginally significant (Table 2). From these two the distance to fences had a negative effect on *C*. *aura* abundance (p = 0.098) and the primary roads a positive effect on the abundance of *G*. *melanoleucus* (p = 0.068). For the rest of the species the strongest effects on the models varied between distance to secondary roads, fences, human settlements and cities (Table 2, Fig. 2). The distance to cities was the strongest predictor variable for *C*. *atratus* (Fig. 2) and also an important variable influencing positively the abundance of individuals in all models except for *V*. *gryphus*. The only variable which had negative effect on the presence of *V*. *gryphus* was the distance to fences (Table 2, Fig. 2). The presence of human settlements had a positive influence in the abundance of *M*. *chimango*, the presence of secondary roads affected in the same way the abundance of *C*. *plancus* (Fig. 2) and also, for both species the closer the city the higher the abundances (Table 2).

**Table 1:** Detection probabilities for all species modeled for the N-mixture approach.

Species	Detection probability	0,025	0,975
*Vultur gryphus*	0,01302	0,00650	0,02589
*Coragyps atratus*	0,01266	0,00614	0,02591
*Cathartes aura*	0,02633	0,01494	0,04601
*Geranoaetus melanoleucus*	0,01495	0,00787	0,02823
*Caracara plancus*	0,01508	0,00762	0,02959
*Milvago chimango*	0,02039	0,01121	0,03680
*Buteo polyosoma*	0,02828	0,00837	0,09115
*Circus cinereus*	0,02046	0,00252	0,14731
*Falco peregrinus*	0,01469	0,00284	0,07228
*Falco femoralis*	0,00822	0,00068	0,09110
*Falco sparverius*	0,00535	0,00160	0,01770
*Parabuteo unicinctus*	-	-	-
*Elanus leucurus*	-	-	-

The first 6 species with lower dispersion were used for the individual analyses, while the others were discarded due the scarce records in our censuses.

**Table 2:** Estimated values obtained from the N-mixture models applied to determine the relationship between the distance to several human constructions and the abundance of the six more abundant species.

	Primary Roads		Secondary Roads		Fences		Human Settlements		Cities	
	Estimate	SE	Estimate	SE	Estimate	SE	Estimate	SE	Estimate	SE
*V*. *gryphus*	-0.515**	0.226	-0.167	0.224	0.607**	**0.249**	0.098	0.169	-0.265	0.172
*C*. *aura*	-0.010	0.426	-0.531	0.544	**0.717**	**0.447**	-0.241	0.341	-0.345	0.396
*C*. *atratus*	-1.048**	0.424	0.132	0.506	-0.036	0.619	0.237	0.293	-1.104***	**0.346**
*G*. *melanoleucus*	-0.295*	**0.161**	-0.155	0.199	-0.163	0.213	0.178	0.137	-0.167	0.122
*M*. *chimango*	0.420	0.309	-0.453	0.405	-0.765	0.491	-0.739***	**0.287**	-0.726**	0.303
*C*. *plancus*	-0.310	0.339	-1.030**	**0.405**	-0.063	0.412	-0.071	0.232	-0.562**	0.270

We present the estimates, standard errors (SE) and the p-value (*p≤ 0.1; **p ≤ 0.05; ***p ≤ 0.01) for every variable in the models. In bold we highlight the most influential variable for each model, which is the one used to show the relationship in the Fig. 2. As the analyses were performed with the distance to the anthropic variables, negative estimate values indicate a positive relationship (and vice versa).

## Discussion

Here we show that human constructions influence the spatial distribution of birds of prey even in a wild and low populated area, in the southern portion of South America. Humanized areas and communication ways positively influenced species richness and individual abundance of several species. Despite we expected some species were negatively influenced by those constructions, the pattern of human environments with an overall positive influence on the biodiversity is consistent with previous studies [55,56].

Most of the species studied appeared to be benefited in relation to the use of anthropic areas. The emerging environments can offer a new source of energy, in an ecosystem that may be naturally poor [57]. Particularly, cities and their surroundings areas are places where there is a new flux of energy available in many forms [5]; for instance, food and wastes in dumps are especially important and can be used mainly by some of the more human-prone scavenger raptors we studied (e.g. *C*. *atratus*). Moreover, those places can also be a source of intermediate disturbance, especially when a gradient-anthropic to natural- is present as it is in our study area, generating habitat and resource diversity which may be used for more species [57]. In the same way, roads provide both energetic resources (road kills) and perching sites (vegetation and fences) [58]. The structures associated with roads (e.g. road sings and poles) and the cleared spaces along them, are used by some species for improving and facilitating their searching and hunting methods [59]. Also the roadside, may favor the occurrence of nesting and perching sites and exotic species as new prey resources, which might positively affect raptor populations [60–63]. However, it is worth to say that the benefits of using human environments can be associated directly with strong adverse effects as contamination, poisoning and road kills as well as electrocution and collision with power lines [12,63].

The fact that we did not find strong negative influences of the presence human constructions on the study species can be because we were not surveying cities, but the surroundings, and that we were mainly counting flying birds. In our censuses we registered every bird seen, no matter the behavior they were displaying. Some species, especially those which we first thought will be away from human activities, have large home ranges and they move several kilometers per day to find food (e.g. the Andean condor [64]). These birds use flight routes that can cross the periphery of a city and several roads, so the records of these raptors flying above human modified environments cannot be interpreted as a proper use of the habitat. In the models condors were positively affected by the distance to primary roads and with approximately the same intensity negatively by the presence of fences. Condors can fly above roads, but ultimately tend to avoid landing close to them for eating [65], while smaller species do not, and tend to be more abundant there [35]. Finally, as we censed the surrounding areas of cities we did not evaluate the major effect of those urban places produce (i.e. [53]). We may expect to find different results whether the survey is done inside a city and if we differentiate what the individual is doing in this area (flying, eating, etc.), since those effects are well known [17,66]. Broader scale studies have found that the majority of the raptor species studied responded as loser species in human modified habitats (e.g. [28]). Then the anthropogenic effects on those top predators and scavengers might be dependent on the scale and the type of sampling, and this should be considered when implementing conservation strategies.

The current scenario of rapid human demographic growth may lead to a change on the raptor assemblage, which is of high concern when this can happen even in wild areas just recently occupied by humans. Medium sized scavenger species (e.g., *C*. *atratus, C*. *plancus* and *M*. *chimango*) actively use urban environments to feed, as the city dumps and their abundance appear to be increasing [30,67].This can lead to a change in the highly nested structure of the carrion usage by scavenger raptors [68], enhancing competition between species and finally displacing the ones that avoid anthropogenic areas to places with less human pressure [69]. Meanwhile, roads, cities and associated infrastructure are increasing rapidly, and fewer natural areas are being available for those species. Moreover, any change on top predators and scavengers, as raptors, are expected to influence different levels of the food chain [70,71]. The modification of species abundances and distribution generated by humans, for instance due to the increase in organic waste, produce changes in the structure of communities and advantages for some species [72]. This should be analyzed in order to know how to equilibrate the new competitive advantages if interventions are needed. This fact has been poorly considered and taking it into account both at local and regional scales and from low to high populated areas will be of help to apply appropriate conservation measures.

## Supporting Information

S1 File Bayesian model fitted to evaluate the effects of the human constructions on the richness of raptor species. (PDF)

S1 Table Results of the Bayesian model used to evaluate the effects of the human constructions on the richness of species of raptors (see the model in S1 File). We present the mean and 95% posterior intervals for the occupancy and the detection probabilities in relation to the presence of human constructions. In bold we highlighted the variables that significantly affected the richness of species accordingly to our model. As the analyses were performed with the distance to the anthropic variables, negative estimate values indicate a positive relationship. We also include here the raw data underlying our work.
(PDF)
