# Supplementary material for: Emerging Ecosystems Change the Spatial Distribution of Top Carnivores Even in Poorly Populated Areas
Source: PLoS One. 2015 Mar 23;10(3):e0118851. doi: 10.1371/journal.pone.0118851 (PMC4370498; doi:10.1371/journal.pone.0118851)
Supplement: S1 File — (PDF) [file pone.0118851.s001.pdf]

Supporting information for:

.

Emerging ecosystems change the spatial distribution of top carnivores even in poorly populated areas

Barbar Facundo, Victoria Werenkraut, Juan Manuel Morales, Sergio Agustín Lambertucci

Laboratorio Ecotono INIBIOMA (CONICET- Universidad Nacional del Comahue).

Address: Quintral 1250, FRF 8400 Bariloche, Río Negro, Argentina. Email: facundo.barbar@gmail.com

S1 File. Bayesian model fitted to evaluate the effects of the human constructions on the richness of raptor species.

```
model {
    # Priors for(k in 1:nspec){
            lpsi[k] ~ dnorm(a, tau1) I(-21,21)
            mu.lp[k] <- b + (rho*sigma2 /sigma1)*(lpsi[k]-a)
            lp[k] ~ dnorm(mu.lp[k], var.lp) I(-21,21)
            bCamino[k] ~ dnorm(mu.bCamino, tau.bCamino) I(-5,5)
            bRuta[k] ~ dnorm(mu.bRuta, tau.bRuta) I(-5,5)
            bAlambrado[k] ~ dnorm(mu.bAlambrado, tau.bAlambrado) I(-5,5)
            bAsenta[k] ~ dnorm(mu.bAsenta, tau.bAsenta) I(-5,5)
            bCiudad[k] ~ dnorm(mu.bCiudad, tau.bCiudad) I(-5,5)
                    }
   # Hyperpriors
            psi.mean ~ dunif(0,1)
            a <- log(psi.mean) - log(1-psi.mean)
```

```
p.mean ~ dunif(0,1)
b <- log(p.mean) - log(1-p.mean)
rho ~ dunif(-1,1)
var.lp <- tau2 /(1.-pow(rho,2))
tau1 <-pow(sigma1,-2)
tau2 <-pow(sigma2,-2)
sigma1 ~ dunif(0,100)
sigma2 ~ dunif(0,100)
tau.bCamino <-pow(sigma.bCamino,-2)
sigma.bCamino ~ dunif(0,5)
mu.bCamino ~ dnorm(0,0.001)
tau.bRuta <-pow(sigma.bRuta,-2)
sigma.bRuta ~ dunif(0,5)
mu.bRuta ~ dnorm(0,0.001)
tau.bAlambrado <-pow(sigma.bAlambrado,-2)
sigma.bAlambrado ~ dunif(0,5)
mu.bAlambrado ~ dnorm(0,0.001)
tau.bAsenta <-pow(sigma.bAsenta,-2)
sigma.bAsenta ~ dunif(0,5)
mu.bAsenta ~ dnorm(0,0.001)
tau.bCiudad <-pow(sigma.bCiudad,-2)
sigma.bCiudad ~ dunif(0,5)
mu.bCiudad ~ dnorm(0,0.001)

# Ecological model for latent occurrence z (process model)
```

```
for(k in 1:nspec) {for (i in 1:nsite)

{ logit(psi[i,k]) <- lpsi[k] + bCamino[k]*dist_camino_st[i] + bRuta[k]*dist_ruta_st [i] + bAlambrado[k]*dist_alambrado_st[i] + bAsenta[k]*dist_asenta_st[i] + bCiudad[k]*dist_ciudad_st[i]  z[i,k] ~ dbern(psi[i,k])}}


# Observation model for observed data Y

for(k in 1:nspec)

{for (i in 1:nsite) { logit(p[i,k]) <- lp[k]     p.eff[i,k] <- z[i,k] * p[i,k]

for (j in 1:nrep){Y[i,j,k] ~ dbern(p.eff[i,k])}}}

# Derived quantities

for(k in 1:nspec)  for (i in 1:nsite) {Nsite[i] <- sum(z[i,])       # Number of occurring species}}
```
