# Supplementary material for: Emerging Ecosystems Change the Spatial Distribution of Top Carnivores Even in Poorly Populated Areas
Source: PLoS One. 2015 Mar 23;10(3):e0118851. doi: 10.1371/journal.pone.0118851 (PMC4370498; doi:10.1371/journal.pone.0118851)
Supplement: S1 Table — We present the mean and 95% posterior intervals for the occupancy and the detection probabilities in relation to the presence of human constructions. In bold we highlighted the variables that significantly affected the richness of species accordingly to our model. As the analyses were performed with the distance to the anthropic variables, negative estimate values indicate a positive relationship. We also include here the raw data underlying our work. (PDF) [file pone.0118851.s002.pdf]

Supporting information for:

.

Emerging ecosystems change the spatial distribution of top carnivores even in poorly populated areas

Barbar Facundo, Victoria Werenkraut, Juan Manuel Morales, Sergio Agustín Lambertucci

Laboratorio Ecotono INIBIOMA (CONICET- Universidad Nacional del Comahue).

Address: Quintral 1250, FRF 8400 Bariloche, Río Negro, Argentina. Email:

facundo.barbar@gmail.com

S1 Table. Results of the Bayesian model used to evaluate the effects of the human constructions on the richness of species of raptors (see the model in File S1).

	Mean	95% posterior interval	
Occupancy	0,7866	0,3312	0,9998
Detection	0,1099	0,0180	0,2503
β1 (primary road)	0,2567	-1,7550	2,3350
β2 (secondary road)	-0,9414	2,5680	0,6209
**β3 (fences)**	**-1,8124**	**-3,5200**	**-0,0214**
β4 (human settlements)	1,4178	-0,2600	3,0670
**β5 (cities)**	**-2,6954**	**-4,5110**	**-0,6610**

We present the mean and 95% posterior intervals for the occupancy and the detection probabilities in relation to the presence of human constructions. In bold we highlighted the variables that significantly affected the richness of species accordingly to our model. As the analyses were performed with the distance to the anthropic variables, negative estimate values indicate a positive relationship.

Lat dec	Long dec	Camino	Ruta	Tendido	Alam	Asent	Ciudad	V_gryp	C_atra	C_aura	G_mela	B_polyo	P_unic	C_cine	P_plan	M_chim	F_pere	F_femo	F_spar
-41,05886	-70,99692	145	0	22	17	492	13635	0	0	0	6	0	0	0	0	0	0	0	0
-41,05886	-70,99692	145	0	22	17	492	13635	0	0	0	0	0	0	0	0	0	0	0	0
-41,05886	-70,99692	145	0	22	17	492	13635	0	0	0	0	0	0	0	0	0	0	0	0
-41,05886	-70,99692	145	0	22	17	492	13635	4	0	0	1	0	0	0	0	0	0	0	0
-41,05886	-70,99692	145	0	22	17	492	13635	0	0	3	0	0	0	0	0	0	0	0	0
-41,05886	-70,99692	145	0	22	17	492	13635	0	0	0	1	0	0	0	0	0	0	0	0
-41,05886	-70,99692	145	0	22	17	492	13635	0	0	0	0	3	0	0	0	0	0	1	0
-41,05886	-70,99692	145	0	22	17	492	13635	2	0	0	0	0	0	0	0	2	0	0	0
-41,05206	-70,98822	792	1137	1114	927	1016	14486	0	0	0	1	0	0	0	0	0	0	0	0
-41,05206	-70,98822	792	1137	1114	927	1016	14486	1	0	0	0	0	0	0	0	0	0	0	0
-41,05206	-70,98822	792	1137	1114	927	1016	14486	0	0	0	0	0	0	0	0	0	0	0	0
-41,05206	-70,98822	792	1137	1114	927	1016	14486	0	0	0	0	0	0	0	0	0	0	0	0
-41,05206	-70,98822	792	1137	1114	927	1016	14486	0	0	0	0	0	0	0	0	0	0	0	0
-41,05206	-70,98822	792	1137	1114	927	1016	14486	0	0	0	0	0	0	0	0	0	0	0	0
-41,05206	-70,98822	792	1137	1114	927	1016	14486	0	0	0	0	0	0	0	0	0	0	0	0
-41,05206	-70,98822	792	1137	1114	927	1016	14486	2	0	0	1	0	0	0	0	0	0	0	0
-41,0455	-70,89767	1594	2090	2068	1703	1887	15228	1	0	0	1	0	0	0	0	0	0	0	0
-41,0455	-70,89767	1594	2090	2068	1703	1887	15228	0	0	0	0	0	0	0	0	0	0	0	0
-41,0455	-70,89767	1594	2090	2068	1703	1887	15228	0	0	0	0	0	0	0	0	0	0	0	0
-41,0455	-70,89767	1594	2090	2068	1703	1887	15228	0	0	0	0	0	0	0	0	0	0	0	0
-41,0455	-70,89767	1594	2090	2068	1703	1887	15228	0	0	0	0	0	0	0	0	0	0	0	0
-41,0455	-70,89767	1594	2090	2068	1703	1887	15228	0	0	0	0	0	0	0	0	0	0	0	0
-41,0455	-70,89767	1594	2090	2068	1703	1887	15228	1	0	0	1	0	0	0	0	0	0	0	0
-41,0455	-70,89767	1594	2090	2068	1703	1887	15228	0	0	0	0	0	0	0	0	0	0	0	0
-41,03214	-70,96506	2915	4050	4000	3709	3810	16885	0	0	0	0	0	0	0	0	0	0	0	0
-41,03214	-70,96506	2915	4050	4000	3709	3810	16885	0	0	0	0	0	0	0	0	0	0	0	0

-41,03214	-70,96506	2915	4050	4000	3709	3810	16885	0	0	1	0	0	0	0	0	0	0	0	0
-41,03214	-70,96506	2915	4050	4000	3709	3810	16885	0	0	0	0	0	0	0	0	0	0	0	0
-41,03214	-70,96506	2915	4050	4000	3709	3810	16885	0	0	0	0	0	0	0	0	0	0	0	0
-41,03214	-70,96506	2915	4050	4000	3709	3810	16885	0	0	0	0	0	0	0	0	0	0	0	0
-41,03214	-70,96506	2915	4050	4000	3709	3810	16885	0	0	0	0	0	0	0	0	0	0	0	0
-41,03214	-70,96506	2915	4050	4000	3709	3810	16885	0	0	0	0	0	0	0	0	0	0	0	0
-41,05886	-70,99692	145	0	22	17	492	13635	0	0	0	0	0	0	0	0	0	0	0	0
-41,05886	-70,99692	145	0	22	17	492	13635	0	0	1	0	0	0	0	0	0	0	0	0
-41,05886	-70,99692	145	0	22	17	492	13635	0	0	0	0	0	0	0	0	0	0	0	0
-41,05886	-70,99692	145	0	22	17	492	13635	0	0	0	1	0	0	0	0	0	0	0	0
-41,06633	-71,00361	911	615	923	622	948	12998	0	0	0	0	0	0	0	0	0	0	0	0
-41,06633	-71,00361	911	615	923	622	948	12998	0	0	0	0	0	0	0	1	0	0	0	0
-41,06633	-71,00361	911	615	923	622	948	12998	1	0	0	1	0	0	0	0	0	0	0	0
-41,06633	-71,00361	911	615	923	622	948	12998	0	0	0	3	0	0	0	1	0	0	0	0
-41,07378	-71,01031	520	1426	516	395	1444	12406	0	0	0	0	0	0	0	0	1	0	0	0
-41,07378	-71,01031	520	1426	516	395	1444	12406	2	0	0	0	0	0	0	0	0	0	0	0
-41,07378	-71,01031	520	1426	516	395	1444	12406	1	0	0	0	0	0	0	0	0	0	0	0
-41,07378	-71,01031	520	1426	516	395	1444	12406	0	0	0	1	0	0	0	1	1	0	0	0
-41,08869	-71,02364	155	3259	854	221	2000	11403	0	0	0	0	0	0	0	0	0	0	0	0
-41,08869	-71,02364	155	3259	854	221	2000	11403	0	0	0	3	0	0	0	1	1	0	0	0
-41,08869	-71,02364	155	3259	854	221	2000	11403	0	0	4	0	0	0	0	0	2	0	0	1
-41,08869	-71,02364	155	3259	854	221	2000	11403	0	0	0	3	0	0	0	0	1	0	0	0
-41,06547	-70,98892	260	0	73	44	1457	14235	0	0	1	3	0	0	0	0	0	0	0	0
-41,06547	-70,98892	260	0	73	44	1457	14235	0	0	0	4	0	0	0	0	0	0	0	0
-41,06547	-70,98892	260	0	73	44	1457	14235	0	0	0	2	0	0	0	0	0	0	0	0
-41,06547	-70,98892	260	0	73	44	1457	14235	1	0	0	2	0	0	0	0	0	0	0	0
-41,06547	-70,98892	260	0	73	44	1457	14235	0	0	0	0	0	0	0	0	1	0	0	0
-41,05939	-70,98158	350	950	878	869	1536	14918	0	0	0	0	0	0	0	0	0	0	0	0
-41,05939	-70,98158	350	950	878	869	1536	14918	0	0	0	0	0	0	0	0	0	0	0	0

-41,05939	-70,98158	350	950	878	869	1536	14918	0	0	0	1	0	0	0	0	0	0	0	0
-41,05939	-70,98158	350	950	878	869	1536	14918	0	0	0	1	0	0	0	0	0	0	0	0
-41,05939	-70,98158	350	950	878	869	1536	14918	4	0	0	0	0	0	0	0	0	0	0	0
-41,05402	-70,97394	1408	1920	1863	1412	1686	15654	0	0	0	0	0	0	0	0	0	0	0	0
-41,05402	-70,97394	1408	1920	1863	1412	1686	15654	0	0	0	0	0	0	0	0	0	0	0	0
-41,05402	-70,97394	1408	1920	1863	1412	1686	15654	0	0	0	2	0	0	0	0	0	0	0	0
-41,05402	-70,97394	1408	1920	1863	1412	1686	15654	0	0	0	0	0	0	0	0	0	0	0	0
-41,05402	-70,97394	1408	1920	1863	1412	1686	15654	0	0	0	0	0	0	0	0	0	0	0	0
-41,03964	-70,95503	315	4153	4063	2464	3309	17523	0	0	0	0	2	0	0	0	0	0	0	0
-41,03964	-70,95503	315	4153	4063	2464	3309	17523	0	0	0	0	1	0	0	0	0	0	0	0
-41,03964	-70,95503	315	4153	4063	2464	3309	17523	0	0	0	0	0	0	0	0	0	0	0	0
-41,03964	-70,95503	315	4153	4063	2464	3309	17523	0	0	0	0	0	0	0	0	0	0	0	0
-41,03964	-70,95503	315	4153	4063	2464	3309	17523	0	0	0	0	0	0	0	0	0	0	0	0
-41,06061	-71,00925	282	0	175	2	242	12599	0	0	0	2	0	0	0	3	0	0	0	0
-41,06061	-71,00925	282	0	175	2	242	12599	1	2	0	0	0	0	0	3	3	0	0	0
-41,06061	-71,00925	282	0	175	2	242	12599	0	0	0	0	0	0	0	0	0	0	0	0
-41,06061	-71,00925	282	0	175	2	242	12599	0	0	0	0	0	0	0	0	5	0	0	0
-41,05389	-71,00139	291	590	472	437	284	13340	0	0	0	0	0	0	0	0	0	0	0	0
-41,05389	-71,00139	291	590	472	437	284	13340	1	0	0	0	0	0	0	0	1	0	0	0
-41,05389	-71,00139	291	590	472	437	284	13340	0	0	0	1	0	0	0	0	0	0	0	1
-41,05389	-71,00139	291	590	472	437	284	13340	0	0	0	0	0	0	0	0	0	0	0	0
-41,04547	-70,99347	681	1515	1510	706	1096	14182	1	0	0	0	0	0	0	0	0	0	0	1
-41,04547	-70,99347	681	1515	1510	706	1096	14182	9	0	0	2	0	0	0	0	0	0	0	0
-41,04547	-70,99347	681	1515	1510	706	1096	14182	0	0	0	0	0	0	0	0	0	0	0	0
-41,04547	-70,99347	681	1515	1510	706	1096	14182	1	0	0	0	0	0	0	0	0	0	0	0
-41,03214	-70,97744	1990	3475	3477	1939	3021	15897	0	0	0	0	0	0	0	0	0	0	0	0
-41,03214	-70,97744	1990	3475	3477	1939	3021	15897	1	0	0	0	0	0	0	0	0	0	0	0
-41,03214	-70,97744	1990	3475	3477	1939	3021	15897	0	0	0	0	0	0	0	0	0	0	0	0
-41,03214	-70,97744	1990	3475	3477	1939	3021	15897	0	0	0	0	0	0	0	0	0	0	0	0

-41,06547	-70,98892	260	0	73	44	1457	14235	0	0	1	0	0	0	0	0	0	0	0	0
-41,06547	-70,98892	260	0	73	44	1457	14235	3	0	1	1	0	0	0	0	0	0	0	0
-41,06547	-70,98892	260	0	73	44	1457	14235	0	0	0	1	0	0	0	0	0	0	0	1
-41,07214	-70,99692	866	1036	1069	844	1841	13534	0	0	0	1	0	0	0	0	0	0	0	0
-41,07214	-70,99692	866	1036	1069	844	1841	13534	2	0	2	4	0	0	0	0	1	0	0	0
-41,07214	-70,99692	866	1036	1069	844	1841	13534	0	0	0	1	0	0	0	1	2	0	0	0
-41,07878	-71,00486	834	1985	1175	113	2195	12874	0	0	0	1	0	0	0	0	0	0	0	0
-41,07878	-71,00486	834	1985	1175	113	2195	12874	2	0	0	3	0	0	0	0	3	0	0	1
-41,07878	-71,00486	834	1985	1175	113	2195	12874	0	0	0	0	0	0	0	0	1	0	0	0
-41,09208	-71,02083	73	3553	1888	112	2885	11692	0	0	0	0	0	0	0	0	0	0	0	0
-41,09208	-71,02083	73	3553	1888	112	2885	11692	0	0	0	0	0	0	0	0	5	0	0	0
-41,09208	-71,02083	73	3553	1888	112	2885	11692	0	0	0	0	0	0	0	0	1	0	0	1
-41,05881	-71,00931	182	0	54	15	148	12599	0	0	0	2	0	0	0	0	0	0	0	0
-41,05881	-71,00931	182	0	54	15	148	12599	0	0	0	1	0	0	0	0	0	0	0	0
-41,05881	-71,00931	182	0	54	15	148	12599	0	0	0	0	0	0	0	0	0	0	0	0
-41,05881	-71,00931	182	0	54	15	148	12599	1	0	0	3	0	0	0	1	0	0	0	0
-41,05881	-71,00931	182	0	54	15	148	12599	1	0	0	1	0	0	0	1	0	0	0	0
-41,06558	-71,01731	51	725	155	50	687	11854	0	0	0	2	0	0	0	0	0	0	0	0
-41,06558	-71,01731	51	725	155	50	687	11854	0	0	0	0	0	0	0	0	0	0	0	0
-41,06558	-71,01731	51	725	155	50	687	11854	0	0	0	0	0	0	0	0	0	0	0	0
-41,06558	-71,01731	51	725	155	50	687	11854	3	0	1	1	0	0	0	0	7	0	0	0
-41,06558	-71,01731	51	725	155	50	687	11854	0	0	5	1	0	0	0	17	6	0	0	0
-41,07217	-71,02533	32	1735	669	468	1064	11145	0	0	0	0	0	0	0	1	12	0	0	0
-41,07217	-71,02533	32	1735	669	468	1064	11145	0	0	0	0	0	0	0	2	0	0	0	0
-41,07217	-71,02533	32	1735	669	468	1064	11145	0	0	1	0	0	0	0	1	1	0	0	0
-41,07217	-71,02533	32	1735	669	468	1064	11145	1	0	0	0	0	0	0	0	5	0	0	0
-41,07217	-71,02533	32	1735	669	468	1064	11145	2	0	0	0	0	0	0	3	6	0	0	0
-41,08497	-71,04156	0	3165	26	25	1885	9858	0	0	0	0	0	0	0	0	0	0	0	0
-41,08497	-71,04156	0	3165	26	25	1885	9858	0	0	0	2	0	0	0	0	1	0	0	0

-41,08497	-71,04156	0	3165	26	25	1885	9858	0	0	0	0	0	0	0	0	0	0	0	0
-41,08497	-71,04156	0	3165	26	25	1885	9858	0	0	1	0	0	0	0	0	0	0	0	0
-41,08497	-71,04156	0	3165	26	25	1885	9858	1	0	0	0	0	0	0	2	0	0	0	0
-41,07192	-70,98056	370	0	68	10	1324	14899	1	0	1	0	0	0	0	0	0	0	0	1
-41,07192	-70,98056	370	0	68	10	1324	14899	0	0	0	0	0	0	0	0	0	0	0	0
-41,06558	-70,98964	234	1071	987	984	597	15621	0	0	0	0	0	0	0	0	0	0	0	0
-41,06558	-70,98964	234	1071	987	984	597	15621	0	0	0	0	0	0	0	0	0	0	0	1
-41,0585	-70,96464	851	2015	1930	595	1043	16344	3	0	0	0	0	0	0	0	0	0	0	1
-41,0585	-70,96464	851	2015	1930	595	1043	16344	0	0	0	0	0	0	0	0	0	0	0	0
-41,04517	-70,94867	2555	4021	3893	1885	2923	17894	1	0	0	0	2	0	0	0	0	0	0	0
-41,04517	-70,94867	2555	4021	3893	1885	2923	17894	0	0	0	0	0	0	0	0	0	0	0	0
-41,05583	-71,02067	676	0	938	7	850	11713	0	0	1	2	0	0	0	1	0	0	0	0
-41,05583	-71,02067	676	0	938	7	850	11713	0	0	0	0	0	0	0	0	1	0	0	0
-41,04867	-71,01261	1060	1010	1180	1012	1270	12558	0	0	0	0	0	0	0	0	1	0	0	0
-41,04867	-71,01261	1060	1010	1180	1012	1270	12558	0	0	0	0	0	0	0	0	0	0	0	0
-41,04256	-71,00442	284	1862	1662	650	1424	13363	0	0	0	0	0	0	0	0	0	0	0	0
-41,04256	-71,00442	284	1862	1662	650	1424	13363	0	0	0	1	0	0	0	0	0	0	0	0
-41,029	-70,98819	1403	3455	3421	2423	3026	15145	1	0	0	0	0	0	0	0	0	0	0	0
-41,029	-70,98819	1403	3455	3421	2423	3026	15145	0	0	0	0	0	0	0	0	0	0	0	0
-41,07197	-70,98064	370	0	68	10	1324	14899	0	0	0	0	0	0	0	0	0	0	0	0
-41,07197	-70,98064	370	0	68	10	1324	14899	0	0	0	0	0	0	0	0	0	0	0	0
-41,07197	-70,98064	370	0	68	10	1324	14899	0	0	0	1	0	0	0	0	0	0	0	0
-41,07197	-70,98064	370	0	68	10	1324	14899	0	0	0	1	0	0	0	1	0	0	0	0
-41,07197	-70,98064	370	0	68	10	1324	14899	0	1	0	0	0	0	0	0	0	0	0	0
-41,07867	-70,97194	231	1024	1078	377	2243	14237	0	0	0	0	0	0	0	0	0	0	0	0
-41,07867	-70,97194	231	1024	1078	377	2243	14237	0	0	0	1	0	0	0	2	0	0	0	0
-41,07867	-70,97194	231	1024	1078	377	2243	14237	3	0	0	0	0	0	0	0	0	0	0	0
-41,07867	-70,97194	231	1024	1078	377	2243	14237	0	0	1	0	0	0	0	0	3	0	0	1
-41,07867	-70,97194	231	1024	1078	377	2243	14237	0	0	0	0	0	0	0	0	0	0	0	2

-41,08533	-70,99661	262	1983	2158	182	3112	13616	0	0	0	0	0	0	0	0	0	0	0	0
-41,08533	-70,99661	262	1983	2158	182	3112	13616	1	0	0	0	0	0	0	0	0	0	0	0
-41,08533	-70,99661	262	1983	2158	182	3112	13616	0	0	0	0	0	0	0	0	0	0	0	0
-41,08533	-70,99661	262	1983	2158	182	3112	13616	1	0	0	0	0	0	0	0	0	0	0	0
-41,08533	-70,99661	262	1983	2158	182	3112	13616	0	0	0	1	0	0	0	2	0	0	0	3
-41,09872	-71,01122	769	3894	2218	764	3933	12628	0	0	0	1	0	0	0	0	0	0	0	0
-41,09872	-71,01122	769	3894	2218	764	3933	12628	0	0	0	1	0	0	0	0	0	0	0	0
-41,09872	-71,01122	769	3894	2218	764	3933	12628	0	0	0	0	0	0	0	0	0	0	0	0
-41,09872	-71,01122	769	3894	2218	764	3933	12628	0	0	0	0	0	0	0	0	0	0	0	1
-41,09872	-71,01122	769	3894	2218	764	3933	12628	0	0	0	4	0	0	1	0	0	0	0	0
-41,05583	-71,02067	694	0	975	5	874	11713	0	0	0	0	0	0	0	0	0	0	0	0
-41,05583	-71,02067	694	0	975	5	874	11713	0	1	1	1	0	0	0	0	0	0	0	0
-41,05583	-71,02067	694	0	975	5	874	11713	0	0	0	1	0	0	0	0	0	0	0	0
-41,05583	-71,02067	694	0	975	5	874	11713	0	0	0	0	0	0	0	0	0	0	0	0
-41,06261	-71,0285	262	620	1122	589	886	10950	0	0	0	3	0	0	0	0	1	0	0	0
-41,06261	-71,0285	262	620	1122	589	886	10950	0	0	0	2	0	0	0	0	0	0	0	0
-41,06261	-71,0285	262	620	1122	589	886	10950	1	0	7	3	0	0	0	0	0	0	0	0
-41,06261	-71,0285	262	620	1122	589	886	10950	0	0	2	0	0	0	0	7	0	0	0	0
-41,06994	-71,03736	77	1450	1272	17	80	10143	0	0	2	0	0	0	0	1	1	0	0	2
-41,06994	-71,03736	77	1450	1272	17	80	10143	0	0	0	0	0	0	0	0	3	0	0	0
-41,06994	-71,03736	77	1450	1272	17	80	10143	0	0	0	0	0	0	0	0	0	0	0	0
-41,06994	-71,03736	77	1450	1272	17	80	10143	0	2	3	0	0	0	0	0	0	0	0	0
-41,08122	-71,05003	220	2955	925	862	1835	9117	0	0	0	2	0	0	0	0	0	2	0	0
-41,08122	-71,05003	220	2955	925	862	1835	9117	0	0	0	3	1	0	0	0	1	0	0	1
-41,08122	-71,05003	220	2955	925	862	1835	9117	0	0	0	0	0	0	0	0	0	0	0	0
-41,08122	-71,05003	220	2955	925	862	1835	9117	1	0	0	0	0	0	0	0	1	0	0	2
-41,07719	-70,97072	660	0	131	10	1145	15734	0	0	0	0	0	0	0	0	0	0	0	0
-41,07719	-70,97072	660	0	131	10	1145	15734	0	0	0	0	0	0	0	0	1	0	0	0
-41,07719	-70,97072	660	0	131	10	1145	15734	0	0	0	2	0	0	0	0	0	0	0	0

-41,07719	-70,97072	660	0	131	10	1145	15734	0	0	0	1	0	0	0	0	0	0	0	0
-41,07108	-70,96281	529	1055	945	919	504	16419	0	0	0	1	0	0	0	0	0	0	0	0
-41,07108	-70,96281	529	1055	945	919	504	16419	4	0	0	0	0	0	0	0	0	0	0	0
-41,07108	-70,96281	529	1055	945	919	504	16419	1	0	0	0	0	0	0	0	0	0	0	0
-41,07108	-70,96281	529	1055	945	919	504	16419	0	0	0	0	0	0	0	0	0	0	0	0
-41,06392	-70,95458	1026	2154	2032	434	1194	17156	0	0	0	0	0	0	0	0	0	0	0	0
-41,06392	-70,95458	1026	2154	2032	434	1194	17156	0	0	0	2	0	0	0	0	0	0	0	0
-41,06392	-70,95458	1026	2154	2032	434	1194	17156	0	0	0	0	0	0	0	0	0	0	0	0
-41,06392	-70,95458	1026	2154	2032	434	1194	17156	0	0	2	1	0	0	0	0	0	0	0	1
-41,05053	-70,93875	2302	4093	3946	2475	3050	18641	0	0	0	0	0	0	0	0	0	0	0	0
-41,05053	-70,93875	2302	4093	3946	2475	3050	18641	0	0	0	0	0	0	0	0	0	0	0	0
-41,05053	-70,93875	2302	4093	3946	2475	3050	18641	0	0	0	0	0	0	0	0	0	0	0	0
-41,05053	-70,93875	2302	4093	3946	2475	3050	18641	0	0	0	0	1	0	0	0	0	0	0	1
-41,05706	-71,03319	570	0	1803	5	1219	10657	0	0	0	1	0	0	0	0	0	0	0	0
-41,05706	-71,03319	570	0	1803	5	1219	10657	2	0	0	0	0	0	0	1	0	0	0	0
-41,05706	-71,03319	570	0	1803	5	1219	10657	0	0	0	2	0	0	0	0	3	0	0	0
-41,04775	-71,02556	1567	958	1806	955	1765	11514	0	0	0	0	0	0	0	0	0	0	0	0
-41,04775	-71,02556	1567	958	1806	955	1765	11514	1	0	0	0	0	0	0	0	0	0	0	0
-41,04775	-71,02556	1567	958	1806	955	1765	11514	0	0	0	0	0	0	0	0	0	0	0	0
-41,04031	-71,01894	1320	1788	2206	1750	2272	12291	7	0	0	2	0	0	0	0	0	0	0	0
-41,04031	-71,01894	1320	1788	2206	1750	2272	12291	0	1	0	1	0	0	0	0	0	0	0	0
-41,04031	-71,01894	1320	1788	2206	1750	2272	12291	4	0	0	3	0	0	0	0	0	0	0	0
-41,02853	-71,00194	937	3379	3280	2143	2749	14226	1	0	0	0	0	0	0	0	0	0	0	0
-41,02853	-71,00194	937	3379	3280	2143	2749	14226	0	0	0	0	0	0	0	0	0	0	0	0
-41,02853	-71,00194	937	3379	3280	2143	2749	14226	1	0	20	0	0	0	0	0	0	0	0	0
-41,07719	-70,97075	660	0	131	10	1145	15734	0	0	0	0	0	0	0	0	0	0	0	0
-41,07719	-70,97075	660	0	131	10	1145	15734	0	0	1	0	0	0	0	0	0	0	0	0
-41,07719	-70,97075	660	0	131	10	1145	15734	0	0	0	0	0	0	0	0	0	0	0	0
-41,08386	-70,97986	194	1039	1203	679	2120	15010	0	0	0	0	1	0	0	2	0	0	0	0

-41,08386	-70,97986	194	1039	1203	679	2120	15010	2	0	0	1	0	0	0	0	0	0	0	0
-41,08386	-70,97986	194	1039	1203	679	2120	15010	0	0	0	0	0	0	0	0	0	0	0	1
-41,09053	-70,98672	249	1926	2147	168	3045	14507	0	0	0	0	0	0	0	0	0	0	0	0
-41,09053	-70,98672	249	1926	2147	168	3045	14507	3	0	0	1	0	0	0	0	0	0	0	0
-41,09053	-70,98672	249	1926	2147	168	3045	14507	0	0	0	2	0	0	0	0	0	0	0	1
-41,10383	-71,00269	1179	4138	3108	1497	4876	13479	0	0	1	0	0	0	0	0	0	0	0	0
-41,10383	-71,00269	1179	4138	3108	1497	4876	13479	3	0	0	0	0	0	0	0	0	0	0	0
-41,10383	-71,00269	1179	4138	3108	1497	4876	13479	0	0	0	2	0	0	0	0	0	0	0	0
-41,05897	-71,03211	570	0	1803	5	1219	10657	0	2	0	0	0	0	0	1	0	0	0	0
-41,05897	-71,03211	570	0	1803	5	1219	10657	0	0	0	0	0	0	0	1	0	0	0	0
-41,05897	-71,03211	570	0	1803	5	1219	10657	0	0	0	2	0	0	1	0	0	1	0	0
-41,05897	-71,03211	570	0	1803	5	1219	10657	8	1	0	1	0	0	0	0	0	0	0	1
-41,06603	-71,04225	101	1118	1790	106	104	9762	0	0	0	0	0	0	0	1	2	0	0	0
-41,06603	-71,04225	101	1118	1790	106	104	9762	0	0	0	0	0	0	0	2	0	0	0	0
-41,06603	-71,04225	101	1118	1790	106	104	9762	0	0	0	0	0	0	0	0	0	0	0	0
-41,06603	-71,04225	101	1118	1790	106	104	9762	1	0	0	2	0	0	0	0	0	0	0	0
-41,07181	-71,05011	1267	2048	1642	303	1251	9093	0	0	0	0	0	0	0	0	1	0	0	0
-41,07181	-71,05011	1267	2048	1642	303	1251	9093	0	0	0	0	0	0	0	0	0	0	0	0
-41,07181	-71,05011	1267	2048	1642	303	1251	9093	0	0	0	0	0	0	0	0	0	0	0	0
-41,07181	-71,05011	1267	2048	1642	303	1251	9093	0	1	0	0	0	0	0	2	0	0	0	1
-41,0865	-71,06536	338	3865	2024	54	3296	8011	0	0	0	0	0	0	0	0	0	0	0	0
-41,0865	-71,06536	338	3865	2024	54	3296	8011	0	0	0	0	0	0	0	0	0	0	0	1
-41,0865	-71,06536	338	3865	2024	54	3296	8011	0	0	0	0	0	0	0	0	0	0	0	0
-41,0865	-71,06536	338	3865	2024	54	3296	8011	0	0	0	0	0	0	0	0	0	1	0	0
-41,08278	-70,96133	580	0	181	20	1755	16537	0	0	0	0	0	0	0	0	0	0	0	0
-41,08278	-70,96133	580	0	181	20	1755	16537	0	0	0	1	0	0	0	0	0	0	0	0
-41,07611	-70,95331	362	958	850	328	1442	17202	0	0	0	0	0	0	0	0	0	0	0	0
-41,07611	-70,95331	362	958	850	328	1442	17202	0	0	0	0	1	0	0	0	0	0	0	0
-41,06939	-70,94536	580	1810	1887	52	1779	17888	0	0	0	0	0	0	0	0	0	0	0	0

-41,06939	-70,94536	580	1810	1887	52	1779	17888	0	0	0	0	0	0	0	0	0	0	0	0
-41,05608	-70,92942	1329	3771	3818	1266	2650	19357	1	0	1	0	1	0	0	0	0	0	0	0
-41,05608	-70,92942	1329	3771	3818	1266	2650	19357	0	0	0	0	0	0	0	0	0	0	0	0
-41,04956	-71,04281	308	0	2895	10	2067	10054	0	0	0	3	0	0	0	0	0	0	0	0
-41,04956	-71,04281	308	0	2895	10	2067	10054	1	0	0	0	0	0	0	0	0	0	0	0
-41,04956	-71,04281	308	0	2895	10	2067	10054	0	0	0	2	0	0	0	0	0	0	0	0
-41,04956	-71,04281	308	0	2895	10	2067	10054	2	17	0	4	0	0	0	0	1	0	0	2
-41,04289	-71,03481	489	982	2757	440	2677	10923	1	0	0	0	0	0	0	0	0	0	0	0
-41,04289	-71,03481	489	982	2757	440	2677	10923	0	0	0	0	0	0	0	0	0	0	0	0
-41,04289	-71,03481	489	982	2757	440	2677	10923	0	0	0	0	0	0	0	0	0	0	0	0
-41,04289	-71,03481	489	982	2757	440	2677	10923	3	2	0	1	0	0	0	0	0	0	0	0
-41,03619	-71,02047	1064	1950	2873	811	2940	11800	0	0	1	0	0	0	0	0	1	0	0	1
-41,03619	-71,02047	1064	1950	2873	811	2940	11800	0	1	0	0	0	0	0	0	1	0	0	0
-41,03619	-71,02047	1064	1950	2873	811	2940	11800	0	0	0	0	0	0	0	0	0	0	0	0
-41,03619	-71,02047	1064	1950	2873	811	2940	11800	1	0	0	0	0	0	0	0	0	0	0	0
-41,02289	-71,01361	352	3749	3868	660	2000	13384	0	0	0	0	0	0	0	0	0	0	0	0
-41,02289	-71,01361	352	3749	3868	660	2000	13384	0	1	0	0	0	0	0	0	0	0	0	0
-41,02289	-71,01361	352	3749	3868	660	2000	13384	0	0	0	0	0	0	0	0	0	0	0	0
-41,02289	-71,01361	352	3749	3868	660	2000	13384	0	0	0	1	0	0	0	0	0	0	0	1
-41,08294	-70,9615	580	0	181	20	1755	16537	0	1	0	0	0	0	0	0	0	0	0	0
-41,08294	-70,9615	580	0	181	20	1755	16537	0	0	0	0	0	0	1	0	0	0	0	0
-41,08294	-70,9615	580	0	181	20	1755	16537	0	0	0	0	0	0	1	0	0	0	0	0
-41,08294	-70,9615	580	0	181	20	1755	16537	0	0	0	0	0	0	0	0	0	0	0	0
-41,08294	-70,9615	580	0	181	20	1755	16537	0	0	0	1	0	0	0	0	0	0	0	0
-41,08294	-70,9615	580	0	181	20	1755	16537	0	0	2	2	0	0	0	0	0	0	0	0
-41,08947	-70,96928	354	1016	1186	918	2418	15955	0	0	0	0	0	0	0	0	0	0	0	0
-41,08947	-70,96928	354	1016	1186	918	2418	15955	0	0	0	0	0	0	0	0	0	0	0	0
-41,08947	-70,96928	354	1016	1186	918	2418	15955	0	0	0	1	0	0	0	0	0	0	1	0
-41,08947	-70,96928	354	1016	1186	918	2418	15955	0	0	0	0	0	0	0	0	0	0	0	0

-41,08947	-70,96928	354	1016	1186	918	2418	15955	0	0	0	0	0	0	0	0	1	0	0	0
-41,08947	-70,96928	354	1016	1186	918	2418	15955	0	0	0	0	0	0	0	0	0	0	0	0
-41,09608	-70,97731	411	2002	2197	895	3276	15390	0	0	0	0	0	0	0	0	0	0	0	0
-41,09608	-70,97731	411	2002	2197	895	3276	15390	0	0	0	1	0	0	0	0	0	0	0	1
-41,09608	-70,97731	411	2002	2197	895	3276	15390	0	0	0	0	0	0	0	0	0	0	0	0
-41,09608	-70,97731	411	2002	2197	895	3276	15390	2	0	0	0	0	0	0	0	0	0	0	1
-41,09608	-70,97731	411	2002	2197	895	3276	15390	3	0	2	0	0	0	0	3	0	0	0	0
-41,09608	-70,97731	411	2002	2197	895	3276	15390	1	0	0	2	0	0	2	0	0	1	0	0
-41,10947	-70,99325	428	3984	4150	2348	4612	14403	0	0	0	0	0	0	0	0	0	0	0	0
-41,10947	-70,99325	428	3984	4150	2348	4612	14403	0	0	0	0	0	0	0	1	0	0	0	1
-41,10947	-70,99325	428	3984	4150	2348	4612	14403	0	0	0	0	0	0	0	0	0	0	0	0
-41,10947	-70,99325	428	3984	4150	2348	4612	14403	1	0	0	1	0	0	0	0	0	0	0	0
-41,10947	-70,99325	428	3984	4150	2348	4612	14403	2	0	0	0	0	0	0	0	0	0	0	0
-41,10947	-70,99325	428	3984	4150	2348	4612	14403	1	1	0	1	0	0	0	1	0	0	0	0
-41,04956	-71,04278	308	0	2895	10	2067	10054	0	0	0	0	0	0	0	0	0	0	0	0
-41,04956	-71,04278	308	0	2895	10	2067	10054	1	0	0	0	0	0	0	0	0	0	0	0
-41,04956	-71,04278	308	0	2895	10	2067	10054	0	0	0	0	0	0	0	1	0	0	0	0
-41,05622	-71,05078	589	1022	3087	275	1770	9239	0	0	0	2	0	0	0	0	0	0	0	0
-41,05622	-71,05078	589	1022	3087	275	1770	9239	1	0	0	0	0	0	0	0	0	0	0	0
-41,05622	-71,05078	589	1022	3087	275	1770	9239	1	0	0	0	0	0	0	1	0	0	0	0
-41,06289	-71,05878	202	2066	2923	283	1635	8485	0	0	0	0	0	0	0	1	0	0	0	0
-41,06289	-71,05878	202	2066	2923	283	1635	8485	0	0	0	1	0	0	0	0	0	0	0	0
-41,06289	-71,05878	202	2066	2923	283	1635	8485	0	0	0	0	0	0	0	0	0	0	0	0
-41,07622	-71,07475	480	2694	3074	523	3433	7099	0	0	0	1	0	0	0	0	0	0	0	0
-41,07622	-71,07475	480	2694	3074	523	3433	7099	0	0	0	1	0	0	0	0	0	0	0	1
-41,07622	-71,07475	480	2694	3074	523	3433	7099	0	0	0	4	0	0	0	0	0	1	0	0
-41,08516	-70,94885	512	0	34	23	3200	17610	0	0	0	2	0	0	0	0	0	0	0	1
-41,08516	-70,94885	512	0	34	23	3200	17610	0	0	0	0	0	0	0	0	0	0	0	1
-41,08516	-70,94885	512	0	34	23	3200	17610	0	0	0	1	0	0	0	0	0	0	0	0

-41,08516	-70,94885	512	0	34	23	3200	17610	0	0	0	0	0	0	1	0	0	0	0	0
-41,07867	-70,94094	430	1079	1011	526	2470	18262	0	0	0	0	0	0	0	0	0	0	0	0
-41,07867	-70,94094	430	1079	1011	526	2470	18262	0	0	0	1	0	0	0	0	0	0	0	0
-41,07867	-70,94094	430	1079	1011	526	2470	18262	0	0	0	0	0	0	0	0	0	0	0	0
-41,07867	-70,94094	430	1079	1011	526	2470	18262	0	0	0	0	0	0	0	0	0	0	0	0
-41,07388	-70,93367	233	1860	1479	273	2846	18865	0	0	0	2	0	0	0	1	0	1	0	0
-41,07388	-70,93367	233	1860	1479	273	2846	18865	0	0	0	0	0	0	0	0	0	0	0	0
-41,07388	-70,93367	233	1860	1479	273	2846	18865	1	0	0	1	0	0	0	0	0	0	0	1
-41,07388	-70,93367	233	1860	1479	273	2846	18865	0	0	0	4	0	0	0	0	0	0	0	0
-41,05815	-70,9174	256	4063	3927	514	1583	20317	0	0	0	0	0	0	0	0	0	0	0	0
-41,05815	-70,9174	256	4063	3927	514	1583	20317	0	0	0	0	0	0	0	1	0	0	0	0
-41,05815	-70,9174	256	4063	3927	514	1583	20317	0	0	0	0	0	0	0	0	0	0	0	0
-41,05815	-70,9174	256	4063	3927	514	1583	20317	0	0	0	0	0	0	0	0	0	0	0	0
-41,04464	-71,05353	537	0	3990	25	2931	9365	1	2	0	0	0	0	0	1	0	0	0	0
-41,04464	-71,05353	537	0	3990	25	2931	9365	0	0	0	0	0	0	0	0	0	0	0	0
-41,04464	-71,05353	537	0	3990	25	2931	9365	0	0	0	0	0	0	0	0	0	0	0	0
-41,04464	-71,05353	537	0	3990	25	2931	9365	1	1	0	5	0	0	0	0	0	0	0	0
-41,04464	-71,05353	537	0	3990	25	2931	9365	0	0	0	0	0	0	0	0	0	1	0	0
-41,03717	-71,04667	606	1008	3897	55	3519	10215	1	0	0	0	0	0	0	4	0	0	0	1
-41,03717	-71,04667	606	1008	3897	55	3519	10215	0	0	2	0	0	0	0	1	1	0	0	0
-41,03717	-71,04667	606	1008	3897	55	3519	10215	0	1	0	0	0	0	0	0	0	0	0	0
-41,03717	-71,04667	606	1008	3897	55	3519	10215	3	0	4	0	0	0	0	0	0	0	1	0
-41,03717	-71,04667	606	1008	3897	55	3519	10215	0	0	0	1	0	0	1	4	0	0	0	0
-41,02972	-71,04025	3	2005	4132	250	3976	11061	2	0	0	0	1	0	0	0	0	0	0	0
-41,02972	-71,04025	3	2005	4132	250	3976	11061	0	0	0	0	0	0	0	0	0	0	0	0
-41,02972	-71,04025	3	2005	4132	250	3976	11061	0	0	0	0	0	0	0	0	0	0	0	1
-41,02972	-71,04025	3	2005	4132	250	3976	11061	1	0	0	1	0	0	0	0	0	0	0	0
-41,02972	-71,04025	3	2005	4132	250	3976	11061	0	0	0	0	0	0	0	0	0	0	0	0
-41,01506	-71,02664	1848	4033	5075	1765	2029	12872	0	0	0	0	0	0	0	0	0	0	0	0

-41,01506	-71,02664	1848	4033	5075	1765	2029	12872	0	0	0	0	0	0	0	0	0	0	0	0
-41,01506	-71,02664	1848	4033	5075	1765	2029	12872	0	0	0	0	0	0	0	0	0	0	0	1
-41,01506	-71,02664	1848	4033	5075	1765	2029	12872	0	0	0	0	0	0	0	0	0	0	0	0
-41,01506	-71,02664	1848	4033	5075	1765	2029	12872	2	0	0	0	0	0	0	0	0	0	0	0
-41,08533	-70,94892	512	0	34	23	3200	17610	0	0	0	1	0	0	0	0	0	0	0	0
-41,08533	-70,94892	512	0	34	23	3200	17610	0	0	0	0	0	0	0	0	0	0	0	0
-41,08533	-70,94892	512	0	34	23	3200	17610	0	0	0	1	0	0	0	0	0	0	0	0
-41,08533	-70,94892	512	0	34	23	3200	17610	0	0	0	3	0	0	0	0	0	0	0	1
-41,09278	-70,95558	991	1095	1081	900	2000	17150	0	0	0	0	0	0	0	0	0	0	0	0
-41,09278	-70,95558	991	1095	1081	900	2000	17150	0	0	0	0	0	0	0	0	0	0	0	0
-41,09278	-70,95558	991	1095	1081	900	2000	17150	0	0	0	0	0	0	0	0	0	0	0	0
-41,09278	-70,95558	991	1095	1081	900	2000	17150	0	0	0	1	0	0	0	0	0	0	0	0
-41,09867	-70,96489	44	1858	2226	191	2435	16479	0	0	0	1	0	0	0	0	0	0	0	0
-41,09867	-70,96489	44	1858	2226	191	2435	16479	0	0	0	0	0	0	0	0	0	0	0	0
-41,09867	-70,96489	44	1858	2226	191	2435	16479	1	0	0	0	0	0	0	0	0	0	0	0
-41,09867	-70,96489	44	1858	2226	191	2435	16479	0	0	0	0	0	0	0	0	0	0	0	0
-41,112	-70,98083	746	3581	3828	1229	3620	15478	1	0	0	0	0	0	0	0	0	0	0	1
-41,112	-70,98083	746	3581	3828	1229	3620	15478	1	0	0	0	0	0	0	0	0	0	1	0
-41,112	-70,98083	746	3581	3828	1229	3620	15478	0	0	0	2	0	0	0	0	0	0	0	0
-41,112	-70,98083	746	3581	3828	1229	3620	15478	0	0	0	0	0	0	0	0	0	0	0	0
-41,04467	-71,05356	537	0	3990	25	2931	9365	0	0	0	0	0	0	0	0	0	0	0	1
-41,04467	-71,05356	537	0	3990	25	2931	9365	0	0	0	0	0	0	0	0	1	0	0	0
-41,05164	-71,06161	692	780	4018	955	2789	8479	0	0	0	0	0	0	0	0	0	0	0	0
-41,05164	-71,06161	692	780	4018	955	2789	8479	1	0	0	0	0	0	0	0	0	0	0	0
-41,05714	-71,06942	350	1216	3097	1192	2995	7710	7	0	0	2	1	0	0	0	0	0	0	0
-41,05714	-71,06942	350	1216	3097	1192	2995	7710	0	0	0	0	0	0	0	0	0	0	0	0
-41,07081	-71,08106	5	1921	3727	362	3109	6531	0	0	0	0	0	0	0	0	0	0	0	0
-41,07081	-71,08106	5	1921	3727	362	3109	6531	0	0	0	0	0	0	0	0	0	0	0	0
